# Comparison of the second-line treatments for patients with small cell lung cancer sensitive to previous platinum-based chemotherapy: A systematic review and Bayesian network analysis

**DOI:** 10.3389/fonc.2023.1154685

**Published:** 2023-03-16

**Authors:** Hekai Shi, Nuojin Guo, Zeming Zhao, Ligang Liu, Tianyi Ni, Jinye Zhang, Yingjie Lu

**Affiliations:** ^1^ Department of Thoracic Surgery, Fudan University Affiliated Huadong Hospital, Shanghai, China; ^2^ Shanghai East Hospital, Tongji University, Shanghai, China; ^3^ Fourth Hospital of Hebei Medical University, Shijiazhuang, China; ^4^ Institute of Therapeutic Innovations and Outcomes, College of Pharmacy, The Ohio State University, Columbus, OH, United States; ^5^ First Hospital of Hebei Medical University, Shijiazhuang, China

**Keywords:** small cell lung cancer, meta-analysis, second-line treatment, relapse, chemotherapy

## Abstract

**Objective:**

It remains unclear what the best second-line treatment is for patients with small-cell lung cancer sensitive to previous platinum-based chemotherapy.

**Methods:**

We systematically screened randomized controlled trials from several online databases. The primary outcome was objective response rate (ORR), and the secondary outcomes were disease control rate (DCR), overall survival (OS), progression-free survival (PFS), and hematological complications graded 3 to 5. The efficacy of included treatments was ranked by surface under the cumulative ranking curve (SUCRA) value.

**Results:**

We included eleven trials involving 1560 patients in quantitative analysis. Triple chemotherapy containing platinum (TP, combination of cisplatin, etoposide, and irinotecan) was associated with favorable ORR (intravenous topotecan vs TP; odds ratio: 0.13, 95% CI:0.03-0.63; SUCRA, 0.94) and PFS (vs intravenous topotecan; hazard ratio, 0.5; 95% CI: 0.25-0.99; SUCRA, 0.90). Belotecan ranked highest for OS (SUCRA, 0.90), while intravenous topotecan plus Ziv-aflibercept ranked highest for DCR (SUCRA, 0.75). TP was more likely to cause anemia and thrombocytopenia while intravenous topotecan plus Ziv-aflibercept resulted in most neutrocytopenia.

**Conclusion:**

TP is the first recommendation for the second-line treatment of sensitive relapsed SCLC. TP achieved priority in ORR and PFS with the most frequent adverse effects in anemia and thrombocytopenia. For patients who cannot tolerate the hematological adverse effects of triple chemotherapy, amrubicin is an optional option. Amrubicin had relatively good ORR and PFS, accompanied by fewer hematological complications. The rechallenge of the platinum doublet is inferior to amrubicin in ORR, DCR, and PFS. Oral topotecan has a similar effect compared with IV topotecan, but oral topotecan was associated with slightly higher safety and less stress in nursing. Belotecan contributed to the best PFS with slightly better safety but was not ideal in other outcomes.

**Systematic review registration:**

https://www.crd.york.ac.uk/PROSPERO/, identifier CRD42022358256.

## 1 Introduction

Lung cancer is one of the leading causes of cancer death, and approximately 15% of all lung cancer diagnoses are small cell lung cancer (SCLC) (1). SCLC is a highly aggressive solid tumor with a poor survival prognosis (2, 3). Although SCLC is sensitive to first-line chemoradiotherapy, many patients still relapsed within one year after previous chemotherapy and died from systematic metastasis. Platinum-based regimens, such as cisplatin/carboplatin plus etoposide/irinotecan are recommended therapy for SCLC as first-line chemotherapy treatment (2). Patients with SCLC are commonly categorized into sensitive or refractory relapse based on whether they responded to first-line chemotherapy. A sensitive relapse is defined as patients who received a complete or partial response during the initial chemotherapy and relapsed more than 60-90 days after first-line chemotherapy. Refractory relapse happens when patients had no response to previous chemotherapy and sustained recurrence within 60-90 days after the completion of initial first-line chemotherapy (4). Patients with refractory relapse might have worsened survival outcomes (5).

Nowadays, the optimal regimen for sensitive relapsed SCLC has not been established (6). Single-agent topotecan was approved by the FDA in the United States in 1996 for the treatment of sensitive relapsed SCLC, while single-agent amrubicin was indicated to be used for relapsed SCLC only in Japan in 2002. Previous meta-analyses suggested that amrubicin provided a higher one-year overall survival rate than topotecan (36% vs 9%) (7, 8). Several studies demonstrated that emerging treatments might be successful for sensitive relapsed SCLC. Immune checkpoint blockade (ICB) targeting programmed cell death protein 1 (PD-1) and cytotoxic T lymphocyte-associated protein 4 (CTLA-4) have been reported to be able to achieve a moderate clinical activity in sensitive relapsed SCLC (9). A large, multicenter, randomized trial in China revealed that niraparib, a poly (adenosine diphosphate-ribose) polymerase (PARP) inhibitor provided better PFS than the placebo in platinum-responsive SCLC (10). However, with the advancement of second-line therapy in patients with sensitive relapsed SCLC, the efficacy and safety of these regimens have not been evaluated and compared systematically.

The existing randomized clinical trials only compared the outcomes of topotecan and amrubicin with other investigational regimens directly. Moreover, the previous meta-analysis included a limited number of regimens, few randomized clinical trials, and they did not compare the outcomes and safety profiles of these regimens in a Bayesian network framework (7, 8). Therefore, we performed this network analysis of RCTs to identify the optimal treatment regimen for patients with sensitive relapsed SCLC in clinical practice and provide robust evidence for clinical decision-making.

## 2 Methods

This network analysis was conducted based on the Preferred Reporting Items for Systematic Reviews and Meta-Analyses (PRISMA) guideline. We registered this project on the PROSPERO website (ID: CRD42022358256) after a preliminary literature search, and all subsequent analyses were performed under the registered protocol.

### 2.1 Search strategy and selection criteria

We systematically searched the Web of Science, Medline, and Embase (Ovid) to screen relevant studies without any date restrictions. Only studies written in English were included. We also carefully read the reference lists of included studies and previous reviews to avoid missing any potential studies. The main search terms and their combinations included SCLC, relapsed, recurrent, second-line treatment, and randomized controlled trial. The detailed literature retrieval strategy was presented in **Table S1 (Supplement)**.

### 2.2 Study selection

The two researchers (Nuojin Guo and Zeming Zhao) identified relevant studies by screening titles and abstracts from the results of a systematic search. Any disputes were resolved through discussion with a third reviewer (Hekai Shi). The inclusion criteria were as follows: (1) head-to-head randomized controlled clinical trials; (2) patients diagnosed with SCLC by histological or cytological laboratory examination; (3) patients sensitive to the first-line platinum-based chemotherapy and relapsed with an interval of at least 60 days after the completion of first-line chemotherapy; (4) patients received the second-line immunotherapy or chemotherapy or their combination to maintain the current status or control the progression of relapsed SCLC; (5) reported at least one following outcomes: objective response rate (ORR), disease control rate (DCR), overall survival (OS), progression-free survival (PFS), and hematological toxic effects (anemia, thrombocytopenia) graded 3 to 5. Studies failing to meet these criteria were excluded.

We defined ORR as the percentage of patients with a complete or partial response, and DCR as the proportion of patients with a complete, partial response, or stable disease after or during the second-line treatment. OS and PFS were defined as the time from randomization to patient death and disease progression, respectively.

### 2.3 Data extraction, risk of bias assessment

Two investigators (Nuojin Guo and Tianyi Ni) extracted the following information from the included studies: name of the first author, publication year, sample size, basic demographic data of the patients, treatment protocol, and efficacy and safety profiles. Treatment-related adverse effects (TRAEs) included thrombocytopenia, anemia, and neutropenia. Any conflicts between the two investigators were resolved through discussion with a third reviewer (Hekai Shi). Four studies did not report basic demographic data for the sensitive relapse population separately, and instead, we extracted corresponding data from the total patients with treated SCLC, regardless of whether they were sensitive to first-line platinum-based chemotherapy (11–14). Most studies provided the survival curve for time-to-event outcomes without the calculated hazard ratios (HRs). We use the method provided by Tierney to fit the survival curve from the original pictures and calculated the estimated HR (15).

Two reviewers (Nuojin Guo and Tianyi Ni) independently evaluated the risk of bias of included studies. The disagreements were consulted with a third person (Hekai Shi). We used the Risk of Bias 2 tool to assess the quality of the randomized trials. ROB2 ranked studies into “low”, “some concerns”, or “high” risk from five bias domains: bias arising from the randomization process, bias due to deviations from intended interventions, bias due to missing outcome data, bias in measurement of the outcome, and bias in the selection of the reported result.

### 2.4 Statistical analysis

We conducted this network meta-analysis using R software (GitHub, San Francisco, US; version 4.1.2) with the “gemtc” package (version 1.0-1). We used odds ratio (OR) and hazard ratio (HR) with 95% confidence interval (95% CI) to calculate the effect size of binary and survival outcomes in the random-effect model. HR for survival outcomes (OS and PFS) and OR for binary outcomes (TRAEs grade 3 to 5) less than 1 and the odds ratio for ORR and DCR greater than 1 were considered to be more favorable. Markov chain Monte Carlo simulation with 20, 000 burn-in and 5, 000 iterations was used to generate the pooled OR and HR. The trace plot, density plot, and Gelman-Rubin plot were used to check the model convergence. The node split method was used to assess the inconsistency between direct and indirect evidence. The efficacy of included regimens was ranked by Surface under the cumulative ranking (SUCRA). The SUCRA value would be 1 if the effect of the agent ranked 1st, and 0 if it ranked the lowest. We used I² statistics to evaluate the size of heterogeneity and if I² > 50%, the heterogeneity is considered significant. P<0.05 (two-tailed) or 95% CI of pooled HR and OR excluding 1 were considered statistically significant in all analyses.

## 3 Results

### 3.1 Selection of studies and risk of bias assessment

After a systematic search, we preliminarily identified a total of 350 eligible studies and finally included 13 trials (11–14, 16–24), and eleven head-to-head with 1560 patients in the quantitative analysis (11, 12, 16–18, 21–24). **Figure S1 (Supplement)** showed the flowchart of the literature search and study determination.

The results of the ROB2 showed that most of the included studies were of moderate and low quality. The most common bias was the bias arising from the randomization process (open-label study design) and bias in the measurement of the outcome (no external independent reviewers to evaluate the results). **Table 1** presented the results of the quality assessment, and detailed information is provided in **Table S2 (Supplement)**.

**Table 1:** Basic characteristics of the included studies.

Study	Treatment	Sample size, (total, female)	Age (median, range)	Country	Current treatment protocol	Treatment cycles (median, range)	Quality assessment
**Inoue 2008 (** 11 **)†**	Amrubicin	29 (5)	70 (54-77)	Japan	40mg/m^2^ on days 1 to 3 every 3 weeks	3 (1-7)	Low risk
	Iv topotecan	30 (5)	64 (32-78)		1.0 mg/m^2^ on days 1 to 5 every 3 weeks	2 (1-4)	
**Inoue 2015 (** 21 **)**	Amrubicin	27 (1)	64 (44–75)	Japan	40mg/m^2^ on days 1 to 3 every 3 weeks	4 (2-8)	Low risk
	Re-challenge of platinum doublet	30 (3)	67 (45–80)		NA	4 (1-7)	
**Jotte 2011 (** 18 **)**	Amrubicin	50 (27)	63 (46-79)	International multi-center	40mg/m^2^ on days 1 to 3 every 3 weeks	6 (1-16)	High risk
	Iv topotecan	26 (15)	68 (46-84)		1.5 mg/m^2^ on days 1 to 5 every 3 weeks	3 (1-16)	
**Eckardt 2007 (** 17 **)**	Iv topotecan	151 (55)	62 (35-82)	International multi-center	1.5 mg/m^2^ on days 1 to 5 every 3 weeks	4	High risk
	Oral topotecan	153 (55)	62.5 (41-82)		2.3 mg/m^2^ on days 1 to 5 every 3 weeks	4	
**von Pawel 2014†** (12)	Amrubicin	424 (180)	62 (22-81)	International multi-center	40mg/m^2^ on days 1 to 3 every 3 weeks	4 (1- 36)	High risk
	Iv topotecan	213 (86)	61 (30-81)		1.5 mg/m^2^ on days 1 to 5 every 3 weeks	4 (1-13)	
**Kang 2021 (** 24 **)**	Iv topotecan	81	NA	South Korea	1.5 mg/m^2^ on days 1 to 5 every 3 weeks	3.7 ± 2.0 (Mean+SD)	High risk
	Belotecan	80	NA		0.5 mg/m^2^ on days 1 to 5 every 3 weeks	4.4 ± 1.9 (Mean+SD))	
**von Pawel 2001** (16)	Iv topotecan	54 (11)	(Mean: 58.2, 35-74)	International multi-center	1.5 mg/m^2^ on days 1 to 5 every 3 weeks	NA	High risk
	Oral topotecan	52 (13)	(Mean: 59.9, 38-79)		2.3 mg/m^2^ on days 1 to 5 every 3 weeks	NA	
**Baize 2020 (** 23 **)**	Oral topotecan	81 (22)	65 (43–81)	France	2.3 mg/m^2^ on days 1 to 5 every 3 weeks	5	High risk
	Re-challenge of platinum doublet	81 (31)	64 (37–84)		Carboplatin on day 1 plus intravenous etoposide (100 mg/m²) from day 1 to day 3 every 3 weeks	3 (1-3)	
**Goto 2016 (** 22 **)**	Iv topotecan	90 (12)	64 (44–75)	Japan	1.0 mg/m^2^ on days 1 to 5 every 3 weeks	NA	High risk
	Cisplatin+etoposide+irinotecan	90 (13)	64 (44-75)		Cisplatin (25 mg/m²) on days 1 and 8, etoposide (60 mg/m²) on days 1-3, irinotecan (90 mg/m²) on day 8 every 14 days	NA	
**Pietanza 2018 (** 13 **)†**	Temozolomide plus veliparib	55 (31)	63 (31-80)	US	Oral placebo 40 mg twice daily on days 1 to 7, and oral Temozolomide 200 mg/m2/day on days 1 to 5 of a 28-day cycle	2 (1-19)	Some concerns
	Temozolomide plus placebo	49 (23)	62 (35 -84)		Oral veliparib 40 mg twice daily on days 1 to 7 and oral Temozolomide 200 mg/m^2^/day on days 1 to 5 of a 28-day cycle	3 (1-21)	
**Pujol 2019 (** 14 **)†**	Carboplatin-etoposide doublet, or oral or Iv topotecan	24 (11)	63.5 (51.8–81.0)	France	carboplatin-etoposide doublet or topotecan 2.3 mg/m^2^ orally or 1.5 mg/m^2^ intravenously on days 1 to 5	NA	High risk
	Atezolizumab	49 (19)	65.9 (51.1–85.5)		1200 mg every 3 weeks	2 (1–24)	
**Allen 2014 (** 19 **)**	Iv topotecan	41(28)	60.1	US	4 mg/m^2^ on days 1, 8, and 15 every 21 days	2 (1-10)	Some concerns
	Iv topotecan plus ziv-aflibercept	42 (25)	63.4		4 mg/m^2^ on days 1, 8 and 15 and ziv-aflibercept 6 mg/kg on day 1 every 21 days	2 (1-13)	
**Evans 2015 (** 20 **)**	Iv topotecan	46	65 (33–80)	International multi-center	1.5 mg/m^2^ on days 1–5 every 21 days	4.0	High risk
	Cabazitaxel	45	62 (40–82)		25 mg/m^2^ on day 1 every 21 days	2.5	

^†^These studies did not report characteristics of patients with sensitive relapse separately, and we extracted corresponding data from the total patients (including sensitive and refractory relapse).

Iv, Intravenous; NA, not available; SD, standard deviation.

### 3.2 Study characteristics

Patients with chemosensitive relapsed SCLC received eight different treatments, including amrubicin, intravenous (IV) topotecan, oral topotecan, re-challenge of platinum doublet (RPD): carboplatin/cisplatin plus etoposide/irinotecan/irinotecan, belotecan, cabazitaxel, ziv-aflibercept plus IV topotecan, and triple chemotherapy containing platinum (TP): the combination of cisplatin, etoposide, and irinotecan. Characteristics of all clinical trials were presented in **Table 1**. Of the 13 studies concerning the efficacy and safety outcomes after the second-line treatment of sensitive relapsed SCLC, four studies were conducted in East Asia, five trials were in Europe and North America, and four were conducted in international multi-center. The proportion of female patients was significantly lower than male patients. Most studies did not restrict third-line therapy for progression after second-line therapy.

### 3.3 Network meta-analyses for outcomes

**Figure 1:**
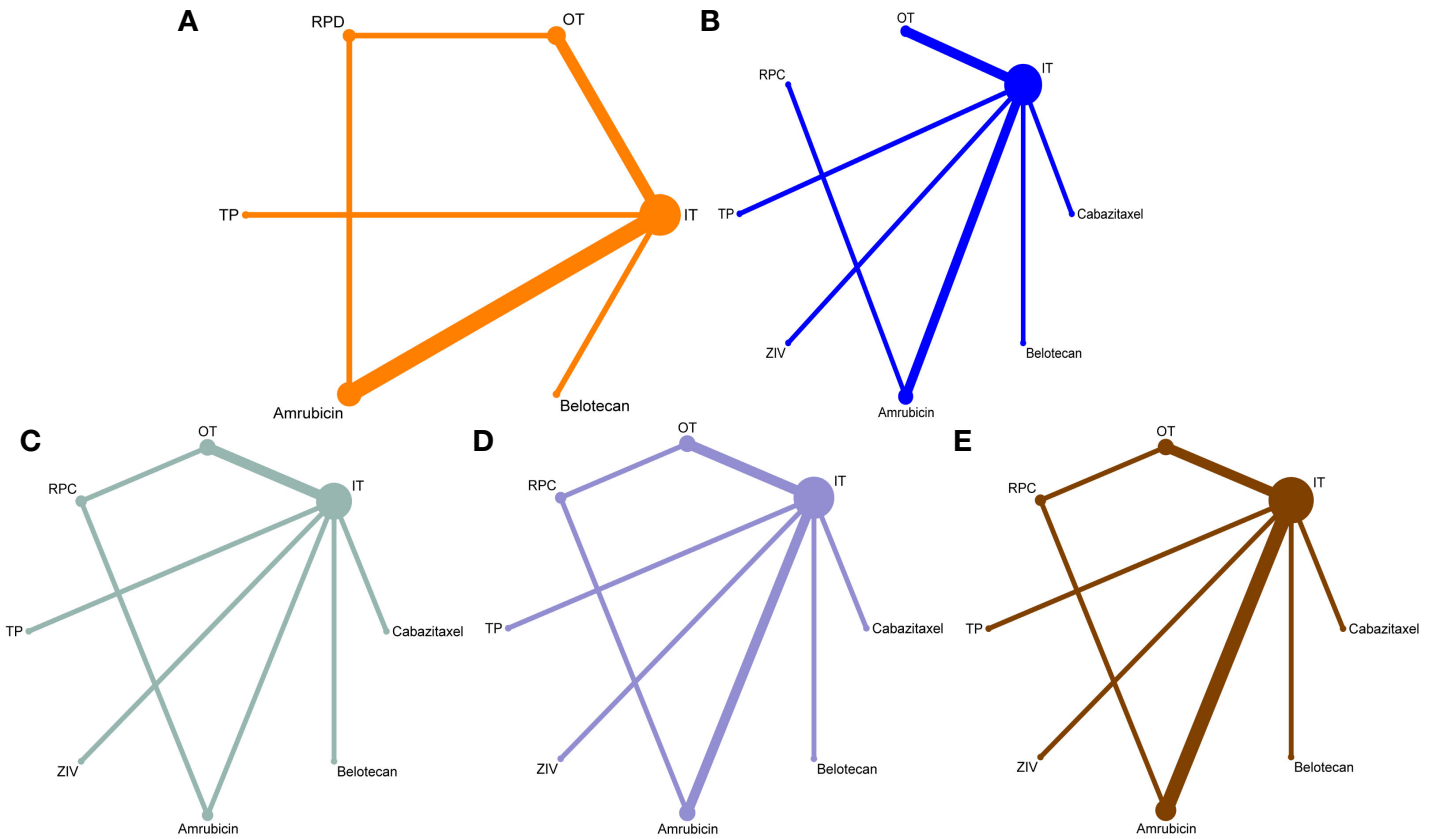
Network maps for the comparisons of the second-line treatments for patients with relapsed small-cell lung cancer sensitive to previous platinum-based chemotherapy. **(A)** for overall response rate, **(B)** for disease control rate, **(C)** for progression-free survival, **(D)** for overall survival rate, and **(E)** for hematological complications grade 3 to 5 (including anemia, thrombocytopenia, and neutropenia). The width of each line represents the number of connections between the two studies, and the size of the dots indicates the frequency of included regimens appeared in the comparison.

The network was designed to allow for multiple comparisons of single regimens and different drugs added to chemotherapy and conventional therapy (**Figure 1**). It included 9 studies for ORR, DCR, and PFS, and 10 studies for OS. The heterogeneity of the included studies was low (for ORR, I^2 =^ 4%).

**Figure 2:**
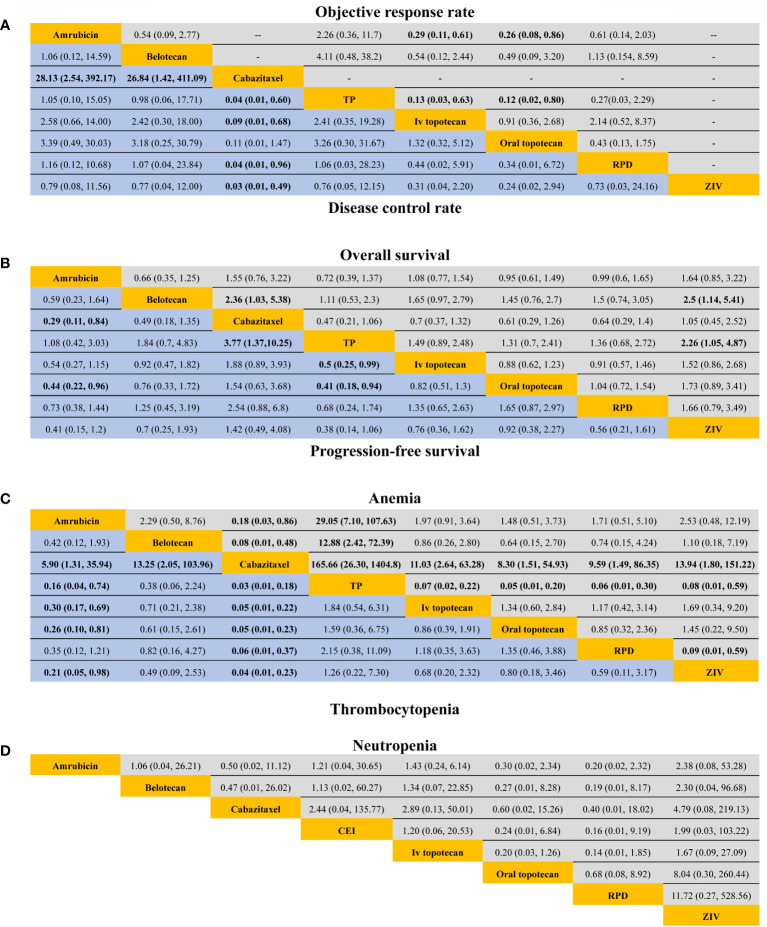
Efficacy and safety profiles in patients sensitive to previous platinum-based chemotherapy. **(A)** Odds ratio and 95% CIs for objective response rate (upper area in grey) and disease control rate (lower area in blue), and an OR > 1.00 indicates a better efficacy. **(B)** Hazard ratios and 95% CIs for overall survival (upper area in grey) and progression-free survival (lower area in blue), and a hazard ratio < 1.00 suggests better survival benefits. **(C, D)** The odds ratio and 95% CIs for hematological complications (anemia, thrombocytopenia, and neutropenia), and an OR < 1.00 indicate better safety.TP, triple therapy containing platinum; RPD, rechallenge of platinum doublet; Ziv, ziv-aflibercept plus intravenous topotecan.

For response outcomes, IV topotecan and oral topotecan have significantly lower ORR (**Figure 2**) compared with amrubicin (odds ratio, 0.29, 95% CI: 0.11-0.61 and 0.26, 95% CI: 0.08-0.86; respectively) and TP (odds ratio; 0.13, 95% CI: 0.03-0.63 and 0.12, 95% CI: 0.02-0.80; respectively). No significant difference was found in the remaining comparison groups. The ORR of cabazitaxel and topotecan plus ziv-aflibercept is not available in network meta-analysis. For DCR, cabazitaxel is significantly lower than all regimens except oral topotecan, and the remaining comparisons provided a similar effect size.

For survival outcomes, only three trials reported PFS benefit (20, 22, 23), and 2 trials report significantly longer OS (22, 24). A significant extended PFS was seen for amrubicin compared with oral topotecan (HR, 0.45; 95% CI: 0.22-0.96) and cabazitaxel (HR, 0.29; 95% CI: 0.11-0.84). PFS in TP was significantly improved versus oral topotecan (HR, 0.41; 95% CI: 0.18-0.93), IV topotecan (HR, 0.5; 95% CI: 0.25-0.99), and cabazitaxel (HR, 0.27; 95% CI: 0.10-0.73).

For OS, cabazitaxel had a shorter OS than Belotecan (HR, 2.36; 95% CI: 1.03-5.38). IV topotecan plus ziv-aflibercept resulted in a worse OS compared with belotecan (HR, 2.5; 95% CI: 1.14-5.41) and TP (HR, 2.26; 95% CI: 1.05-4.87). No particular superiority in the prolongation of OS was observed among the remaining treatments, with the HR close to 1.

### 3.4 Network meta-analyses for TRAEs of grade 3 or greater

Eleven studies reported TRAE grades 3 to 5. Only hematological complications were included in this network meta-analysis because all-cause non-hematological adverse events were rare. Four studies did not report TRAEs for patients with sensitive relapse separately, and we adopted corresponding safety profiles from the total patients (including sensitive and refractory relapse) (11, 12, 19, 20). Cabazitaxel had a significantly lower incidence of anemia and thrombocytopenia than the remaining treatments. TP was more likely to cause anemia than other treatments. Amrubicin was associated with a lower incidence of thrombocytopenia compared with cisplatin plus etoposide plus irinotecan, IV topotecan, and oral topotecan. No significant difference was observed between included studies for the incidence of neutropenia.

### 3.5 Rank probability and inconsistency assessment

**Figure 3:**
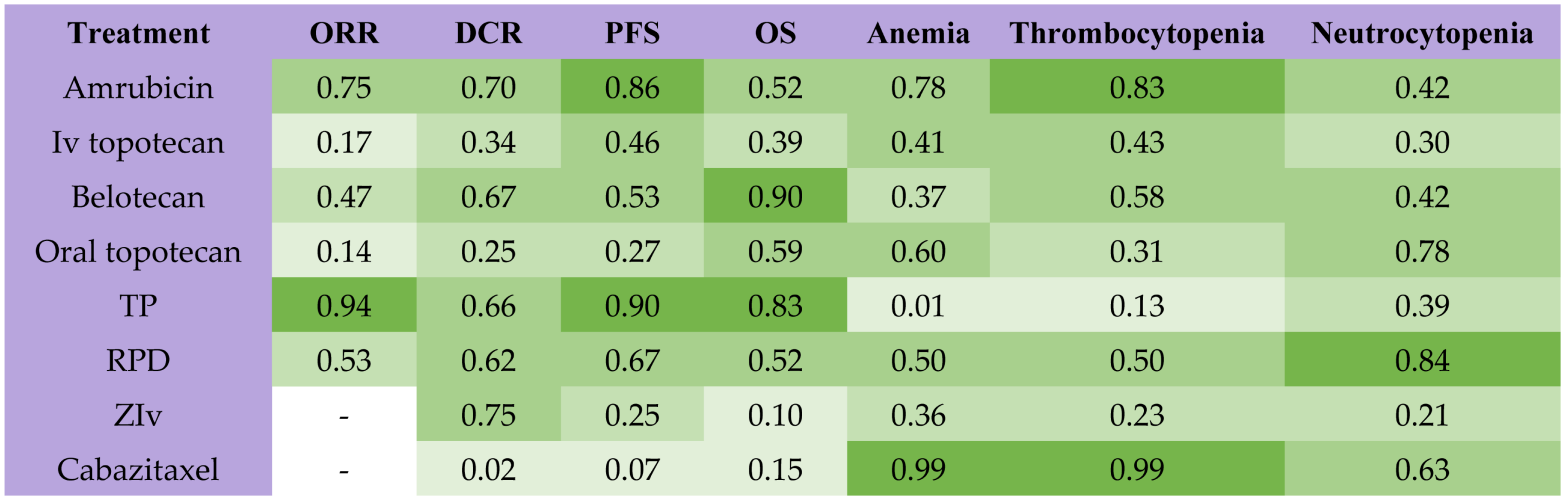
Rank probabilities with SUCRA value for different outcomes in 8 kinds of second-line treatments for patients sensitive to previous platinum-based chemotherapy. The deeper green color indicates more favorable outcomes. SUCRA, Surface under the cumulative ranking curve; ORR, objective response rate; DCR, disease control rate; OS, overall survival; PFS, progression-free survival; TP, TP, triple therapy containing platinum; RPD, rechallenge of platinum doublet; ZIv, ziv-aflibercept plus intravenous topotecan.

Among all second-line treatments for patients with SCLC sensitive to previous chemotherapy, TP had the highest probability of ranking first for ORR and PFS, while amrubicin had the highest probability of ranking first for DCR (**Figure 3**). Belotecan (SUCRA, 0.90) and cisplatin plus etoposide plus irinotecan (SUCRA,0.83) contributed to the highest possibility for OS. Cabazitaxel provided the lowest probability of ranking first for anemia (SUCRA, 0.99) and thrombocytopenia (SUCRA, 0.99), while the re-challenge of platinum doublet had the lowest probability of ranking first for neutrocytopenia (SUCRA, 0.84). The results of the node split method indicated that no significant inconsistency was observed in direct, indirect, and overall effects (**Table S3, Supplement**). The result of the node split method in DCR was missing because there was no closed loop for network comparison.

## 4 Discussion

This bayesian network meta-analysis included 13 randomized clinical trials for systematical review and eleven head-to-head studies including 1560 patients in quantitative analysis. Moreover, we compared the efficacy and safety of multiple second-line treatments for patients with chemosensitive SCLC. The pooled results suggested that the triple chemotherapy containing platinum (TP) was associated with favorable ORR and PFS and provided relatively good effects in OS and DCR. However, TP was more likely to cause serious hematological complications, such as anemia and thrombocytopenia. Oral topotecan and IV topotecan provided a poor effect in all outcome measures, while IV topotecan seemed to cause more adverse events including anemia and neutropenia than oral topotecan. Re-challenge of platinum doublet achieved a moderate effect in improving all outcomes with moderate frequency in TRAEs.

Repeated use of first-line regimens is one of the most common treatments for patients with sensitive recurrent SCLC. The previous meta-analysis showed that the re-challenge group had a significantly longer median PFS than non-platinum-based chemotherapy in sensitive relapsed SCLC (25). In this network analysis, we observed relatively high proportions of patients with sensitive relapsed SCLC receiving a response to re-challenge of the platinum doublet. This phenomenon appears to be the residual chemical sensitivities of tumor cells that were not eliminated by the initial treatment (16, 26). Some scholars suggested that the efficacy of TP (cisplatin plus etoposide and irinotecan) might also be partly associated with the alteration of cisplatin plus etoposide and cisplatin plus irinotecan in their initial first-line treatment (22).

Topotecan, with specific targeting to DNA topoisomerase-I, provided relatively poor effect sizes in nearly all outcome measures in this network meta-analysis. This analysis indicated that significantly better ORR was observed in amrubicin and TP compared with oral and IV topotecan. A similar response rate, survival outcomes, and safety profiles were observed between oral and IV topotecan. Therefore, compared with IV topotecan, oral topotecan may offer a useful, and convenient treatment option for patients in a palliative setting. Belotecan, a new camptothecin analogue topoisomerase I inhibitor, was approved as the third-line treatment for metastatic SCLC by FDA (24), which contributed to the favorable OS in this network meta-analysis. The researchers attribute favorable OS to a low incidence of adverse effects because patients in the belotecan group received nearly one more treatment cycle than the topotecan group on average with similar incidences of AEs (24).

Amrubicin is a fully synthetic 9-aminoanthracycline. Our results showed that amrubicin may be superior to IV topotecan in all outcome measures, with acceptable toxicity for patients with relapsed SCLC. Amrubicin may cause high frequency in neutropenia. However, a reduction of the initial dose and preventive use of colony-stimulating factors may reduce the frequency of febrile neutropenia (27, 28).

Ziv-aflibercept is an antibody to vascular endothelial growth factor (VEGF). A high amount of circulating VEGF in SCLC was linked to poor survival and response profiles (29). In this network meta-analysis, we found IV topotecan plus ziv-Aflibercept performed well only on DCR. Cabazitaxel, a second-generation taxane, showed antitumor activity in other refractory solid tumors (30). Cabazitaxel provided the best safety outcomes in anemia and thrombocytopenia but was less effective than IV topotecan.

Since second-line treatments for patients with relapsed small-cell lung cancer are more of are palliative treatment, a tolerable toxicity profile is essential. In this meta-analysis, we found that severe adverse events were more common in the combined treatment group than in the single regimens group. The relationship between complications and survival outcomes remains controversial. A previous study suggested that anemia was a risk factor for poor prognosis of chemotherapy, and patients with NSCLC without baseline anemia are significantly associated with longer survival outcomes in post-chemotherapy (31). However, another retrospective study with a large sample size found no association between anemia and OS for SCLC (32). In this network meta-analysis, we found that patients who received a triple combination of platinum-based chemotherapy reported more hematological complications such as anemia and thrombocytopenia but provided relatively higher PFS and OS.

In our study, all patients were sensitive to platinum-based chemotherapy during the first-line treatment. However, SCLC would become resistant to second-line treatments eventually, with PFS less than one year in most studies. The mechanisms that lead to chemoresistance in SCLC are not fully understood. Drug resistance might be achieved through DNA damage repair, altered differentiation state, altered tumor metabolism, and microenvironment changes (26).

Immunotherapy for relapsed SCLC achieved moderate antitumor activity with significantly fewer hematological complications (9, 33). However, immunotherapy was not currently eligible for quantitative analysis in this network analysis because of a lack of connecting arms. Nivolumab and pembrolizumab are fully human IgG4 PD-1 ICB antibodies, and ipilimumab is a fully human IgG1 CTLA-4 ICB antibody. In CheckMate 032, an open-label, multicenter RCT, the 1-year OS rate for nivolumab or nivolumab plus ipilimumab was similar to the second-line topotecan or amrubicin reported in previous trials for recurrent SCLC (33). CheckMate 331, another open-label RCT (569 patients), indicated that nivolumab provided similar survival outcomes compared with chemotherapy (amrubicin or topotecan) (9). Atezolizumab, an engineered programmed cell death ligand 1 (PD-L1) antibody, provided superiority in PFS compared with topotecan or carboplatin-etoposide in relapsed SCLC in a randomized phase II trial involving 73 patients (14). Results from two basket trials (KEYNOTE-028 and KEYNOTE-158) suggested that pembrolizumab exhibited a moderate ORR (19.3%, 95% CI: 11.4-29.4%) in patients with recurrent or metastatic SCLC after two or more lines therapy (34).

Targeted therapies for relapsed SCLC are still a challenge. In most cases, the efficacy of targeted therapy is similar to or slightly worse than conventional chemotherapy, with a similar probability of complications (35, 36). Alisertib (MLN8237) is an investigational Aurora A kinase (AAK) inhibitor. AAK inhibitor is a key regulator of mitosis. Inhibition of AAK may lead to mitosis suspension, inducing cell apoptosis (37). A randomized phase II study indicated that paclitaxel plus alisertib and paclitaxel plus placebo demonstrated similar median PFS was seen among in relapsed SCLC (HR, 0.77; 95% CI:0.56-1.01) (38). Dinutuximab, an anti-disialoganglioside antibody, may suppress the proliferation of GD2-positive SCLC cells, induce apoptosis and enhance the apoptotic effects of chemotherapy. However, in a phase 3 RCT with 471 patients, dinutuximab plus irinotecan did not improve survival and response outcomes compared with topotecan or irinotecan in relapsed SCLC (36).

Olaparib and veliparib are PARP inhibitors. A randomized trial (220 patients) performed in the United Kingdom showed that olaparib did not significantly alter PFS and OS compared with the placebo (39). Adding veliparib into cisplatin and etoposide in the first-line treatment of SCLC had no significant effect on survival outcomes (40). In a double-blind, phase II RCT involving 104 patients, the ORR for veliparib plus temozolomide (an oral alkylating agent) was 41% vs 11% for temozolomide plus placebo in sensitive relapsed SCLC (13). SLFN11, a protein at the S-phase checkpoint, causes cell cycle arrest and apoptosis of replication-stressed cells under the DNA-damaging agents (41). Patients with SLFN11-positive tumors treated with temozolomide plus veliparib had significantly extended PFS and OS compared with temozolomide plus placebo (13). Linsitinib (OSI-906) is an insulin growth factor-1 receptor (IGF-1R) targeting agent. In a randomized phase II study involving 37 patients, no clinical activity was seen for linsitinib compared with IV topotecan in patients with relapsed SCLC (35).

Lurbinectedin may become the new standard second-line regimen for SCLC. As a novel RNA-polymerase-II inhibitor, lurbinectedin suppresses the DNA damage repair in tumor cells and alters the tumor microenvironment. A Phase I study with 27 patients found that lurbinectedin plus doxorubicin contributed to over 90% ORR (10 from 12 patients) in sensitive relapsed SCLC (42). In an open-label, single-arm trial for lurbinectedin involving 105 relapsed SCLC patients, ORR for lurbinectedin in sensitive relapsed SCLC was significantly higher than refractory SCLC with acceptable tolerance (45% vs 22%) (43). But further randomized controlled trials are needed to confirm its efficacy.

Another strategy being investigated to improve SCLC patient outcomes is combined chemotherapy, immunotherapy, and targeted therapy. A previous meta-analysis involving 1553 patients from 4 RCTs reported that adding a PD-1/D-L1 ICB to chemotherapy led to a significant benefit in OS, ORR, and PFS in the first-line treatment of SCLC (44). Zou et al. performed a network meta-analysis and suggested that PD-L1 inhibitor (atezolizumab and durvalumab) plus etoposide-platinum provided the best OS and DCR as the first-line treatment for extensive-stage SCLC (45). However, only limited long-term data support the treatment landscape for combining immunotherapy or targeted therapy with chemotherapy for patients with relapsed SCLC.

The study has some limitations. First, many of the included studies were open-label trials, which could create potential bias. Second, the number of studies included and the sample size of patients were relatively small, and more clinical trials are needed to produce more reliable results. Third, due to limited data on individual patients, we were unable to provide subgroup analyses by stratifying patients by sex, age, smoking status, or other factors that might be associated with outcome measures. Forth, some emerging therapies have not been included because of a lack of connecting comparisons. Fifth, the quality of the included studies was relatively low, and most of the studies had a moderate or high bias.

Overall, there are limited treatment options for patients with relapsed SCLC. For patients with chemotherapy-refractory relapsed SCLC, just a few treatment regimens achieved a response rate greater than 10% (12, 20). However, for sensitive relapsed SCLC, those agents in most studies have demonstrated response rates of more than 20% and even achieved more than 50% in combination with the re-challenge of platinum doublet (11, 22). Although topotecan is considered the standard second-line treatment for relapsed SCLC, we found many alternative regimens in this network analysis. TP is the first recommendation for the second-line treatment of sensitive relapsed SCLC. TP achieved priority in ORR and PFS with the most frequent adverse effects in anemia and thrombocytopenia. For patients who cannot tolerate the hematological adverse effects of triple chemotherapy, amrubicin is an optional option. Amrubicin had relatively good ORR and PFS, accompanied by fewer hematological complications. The rechallenge of the platinum doublet is inferior to amrubicin in ORR, DCR, and PFS. Oral topotecan has a similar effect compared with IV topotecan, but oral topotecan was associated with slightly higher safety and less stress in nursing. Belotecan contributed to the best PFS with slightly better safety but was not ideal in other outcomes.

## Data availability statement

Publicly available datasets were analyzed in this study. This data can be found here: Data proceed in this meta-analysis are available in Science Data Bank (URL: https://www.scidb.cn/s/jqmi2q).

## Author contributions

Conceptualization, HS and NG; methodology, HS; validation, ZZ, LL, and TN; formal analysis, HS and NG; investigation, ZZ, TN, JZ, and NG; data curation, NG, ZZ, JZ, and TN; writing—original draft preparation, HS and LL; writing—review and editing, YL; visualization, HS; supervision, YL; project administration, YL. All authors contributed to the article and approved the submitted version.
