# Supplementary material for: Comparison of the second-line treatments for patients with small cell lung cancer sensitive to previous platinum-based chemotherapy: A systematic review and Bayesian network analysis
Source: Front Oncol. 2023 Mar 16;13:1154685. doi: 10.3389/fonc.2023.1154685 (PMC10061131; doi:10.3389/fonc.2023.1154685)
Supplement: Supplementary file 1 [file DataSheet_1.docx]

**Table S1.** **Literature Search Strategy**

Table 1a. Medline

| NO | keywords | results |
| --- | --- | --- |
| #1 | **(small cell lung cancer) OR (SCLC)** | 111,995 |
| #2 | **(non-small) OR (NSCLC)** | 95,972 |
| #3 | **((relapsed) OR (recurrent)) OR (second-line) OR (after chemotherapy)** | 1,849,075 |
| #4 | **(randomized controlled trial) OR (RCT)** | 777,430 |
|  | #1 NOT #2 AND #3 AND #4 | 427 |

Table 1b. Web of Science

| NO | keywords | results |
| --- | --- | --- |
| #1 | (((TS=((second-line))) OR TS=(relapsed)) OR TS=(recurrent)) OR TS=((after chemotherapy)) | 848,605 |
| #2 | ((small cell lung cancer)) OR (SCLC) | 215,137 |
| #3 | (relapsed or recurrent or second-line or after chemotherapy).mp | 769013 |
| #4 | TS=(randomized controlled trial) OR TS=(RCT) | 713,496 |
| #6 | #1 AND #2 NOT #3 AND #4 | 327 |

**Table 1c. Embase**

| NO | keywords | results |
| --- | --- | --- |
| #1 | lung small cell cancer/ or small cell lung cancer/ or small cell carcinoma | 43633 |
| #2 | lung non small cell cancer/ or non small cell lung cancer | 148487 |
| #3 | (TS=((non-small))) OR TS=(NSCLC) | 178,225 |
| #4 | TS=(randomized controlled trial) OR TS=(RCT) | 726,924 |
| #6 | #1 AND #2 NOT #3 AND #4 | 194 |

**Table S2. Results of quality assessment**

| **Study** | **Bias arising from the randomization process** | **Bias due to deviations from intended interventions** | **Bias due to missing outcome data** | **Bias in measurement of the outcome** | **Bias in selection of the reported result** |
| --- | --- | --- | --- | --- | --- |
| Inoue 2008 | Low | Low | Low | Low | Low |
| Inoue 2015 | Low | Low | Low | Low | Low |
| Jotte 2011 | High | Low | Low | No information | Low |
| Eckardt 2007 | High | Low | Some concerns | No information | Low |
| Pawel 2014 | High | Some concerns | Low | No information | Low |
| Kang 2021 | High | Low | Low | Low | Low |
| Pawel 2001 | No information | High | Low | Low | Low |
| Baize 2020 | High | No information | Low | Low | Low |
| Goto 2016 | High | Low | Low | No information | Low |
| Pietanza 2018 | Low | Low | Some concerns | No information | Low |
| Pujol 2019 | High | Low | Low | No information | Low |
| Allen 2014 | No information | No information | Low | No information | Low |
| Evans 2015 | High | Low | Some concerns | No information | Low |

**Table S3. Results of the node split method**

| **Overall response rate** | | | |
| --- | --- | --- | --- |
| **Treatment** | **Odds ratio** | | **P-value** |
| IV topotecan  vs Amrubicin | direct | 0.33 (0.10,0.83) | 0.37 |
|  | indirect | 0.11 (0.01, 1.83) |  |
|  | network | 0.29 (0.10, 0.64) |  |
| Re-challenge of platinum doublet vs amrubicin | direct | 0.37 (0.06, 2.37) | 0.36 |
|  | indirect | 1.09 (0.09, 0.64) |  |
|  | network | 0.60 (0.14, 1.99) |  |
| Oral topotecan vs Iv topotecan | direct | 1.03 (0.32, 3,96) | 0.36 |
|  | indirect | 0.36 (0.03, 6.15) |  |
|  | network | 0.90 (0.40, 2.50) |  |
| Re-challenge of platinum doublet vs oral topotecan | direct | 3.10 (0.59, 17.4) | 0.37 |
|  | indirect | 1.04 (0.09, 13.4) |  |
|  | network | 2.30 (0.64, 8.37) |  |
| **Overall survival** | | | |
| **Treatment** | **Odds ratio** | | **P-value** |
| IV topotecan  vs Amrubicin | direct | 1.1 (0.71, 1.6) | 0.85 |
|  | indirect | 1.2 (0.42, 3.5) |  |
|  | network | 1.1 (0.77, 1.6) |  |
| Re-challenge of platinum doublet vs amrubicin | direct | 1.1 (0.44, 2.5) | 0.86 |
|  | indirect | 0.96 (0.44, 2.1) |  |
|  | network | 0.99 (0.60, 1.7) |  |
| Oral topotecan vs Iv topotecan | direct | 0.87 (0.57, 1.3) | 0.86 |
|  | indirect | 0.95 (0.33, 2.7) |  |
|  | network | 0.88 (0.61, 1.2) |  |
| Re-challenge of platinum doublet vs oral topotecan | direct | 1.0 (0.62, 1.7) | 0.85 |
|  | indirect | 1.1 (0.40, 3.2) |  |
|  | network | 1.0 (0.71, 1.6) |  |
| **Progression-free survival** | | | |
| **Treatment** | **Odds ratio** | | **P-value** |
| IV topotecan  vs Amrubicin | direct | 1.5 (0.57, 4.1) | 0.58 |
|  | indirect | 2.3 (0.62, 8.3) |  |
|  | network | 1.8 (0.86, 3.7) |  |
| Re-challenge of platinum doublet vs amrubicin | direct | 1.5 (0.63, 3.6) | 0.59 |
|  | indirect | 1.0 (0.26, 4.0) |  |
|  | network | 1.4 (0.69, 2.7) |  |
| Oral topotecan  vs Iv topotecan | direct | 1.2 (0.66, 2.1) | 0.58 |
|  | indirect | 1.7 (0.38, 7.9) |  |
|  | network | 1.2 (0.76, 2.0) |  |
| Re-challenge of platinum doublet vs oral topotecan | direct | 0.57 (0.26, 1.3) | 0.59 |
|  | indirect | 0.84 (0.20, 3.5) |  |
|  | network | 0.61 (0.33, 1.2) |  |
| **Anemia** | | | |
| **Treatment** | **Odds ratio** | | **P-value** |
| IV topotecan  vs Amrubicin | direct | 1.97 (0.72, 4.59) | 0.87 |
|  | indirect | 1.61 (0.11, 21.8) |  |
|  | network | 1.96 (0.89, 3.91) |  |
| Re-challenge of platinum doublet vs amrubicin | direct | 1.53 (0.23, 10.8) | 0.85 |
|  | indirect | 1.94 (0.21, 14.3) |  |
|  | network | 1.74 (0.49, 5.59) |  |
| Oral topotecan  vs Iv topotecan | direct | 0.78 (0.30, 2.37) | 0.82 |
|  | indirect | 0.60 (0.05, 10.2) |  |
|  | network | 0.76 (0.35, 1.87) |  |
| Re-challenge of platinum doublet vs oral topotecan | direct | 1.24 (0.26, 5.41) | 0.85 |
|  | indirect | 1.01 (0.10, 11.9) |  |
|  | network | 1.17 (0.38, 3.30) |  |
| **Thrombocytopenia** | | | |
| **Treatment** | **Odds ratio** | | **P-value** |
| IV topotecan  vs Amrubicin | direct | 3.39 (1.27, 6.56) | 0.7 |
|  | indirect | 2.27 (0.23, 23.4) |  |
|  | network | 3.35 (1.44, 5.96) |  |
| Re-challenge of platinum doublet vs amrubicin | direct | 2.26 (0.38, 15.1) | 0.76 |
|  | indirect | 3.25 (0.40, 19.0) |  |
|  | network | 2.81 (0.81, 8.34) |  |
| Oral topotecan  vs Iv topotecan | direct | 1.20 (0.43, 2.91) | 0.76 |
|  | indirect | 0.85 (0.10, 10.3) |  |
|  | network | 1.15 (0.57, 2.49) |  |
| Re-challenge of platinum doublet vs oral topotecan | direct | 0.79 (0.22, 3.01) | 0.77 |
|  | indirect | 0.57 (0.07, 5.34) |  |
|  | network | 0.72 (0.28, 1.97) |  |
| **Neutrocytopenia** | | | |
| **Treatment** | **Odds ratio** | | **P-value** |
| IV topotecan  vs Amrubicin | direct | 1.20 (0.14, 7.37) | 0.59 |
|  | indirect | 4.11 (0.02, 1.19e+03) |  |
|  | network | 1.41 (0.25, 6.30) |  |
| Re-challenge of platinum doublet vs amrubicin | direct | 0.31 (0.01, 9.35) | 0.59 |
|  | indirect | 0.0961 (0.01, 6.19) |  |
|  | network | 0.20 (0.01, 2.12) |  |
| Oral topotecan  vs Iv topotecan | direct | 0.173 (0.01, 1.31) | 0.58 |
|  | indirect | 0.55 (0.01, 121.0) |  |
|  | network | 0.208 (0.03, 1.29) |  |
| Re-challenge of platinum doublet vs oral topotecan | direct | 0.490 (0.02, 13.0) | 0.6 |
|  | indirect | 1.50 (0.02, 181.0) |  |
|  | network | 0.65 (0.06, 8.42) |  |

**Figure S1. Flowchart of Study Selection and Design**

| 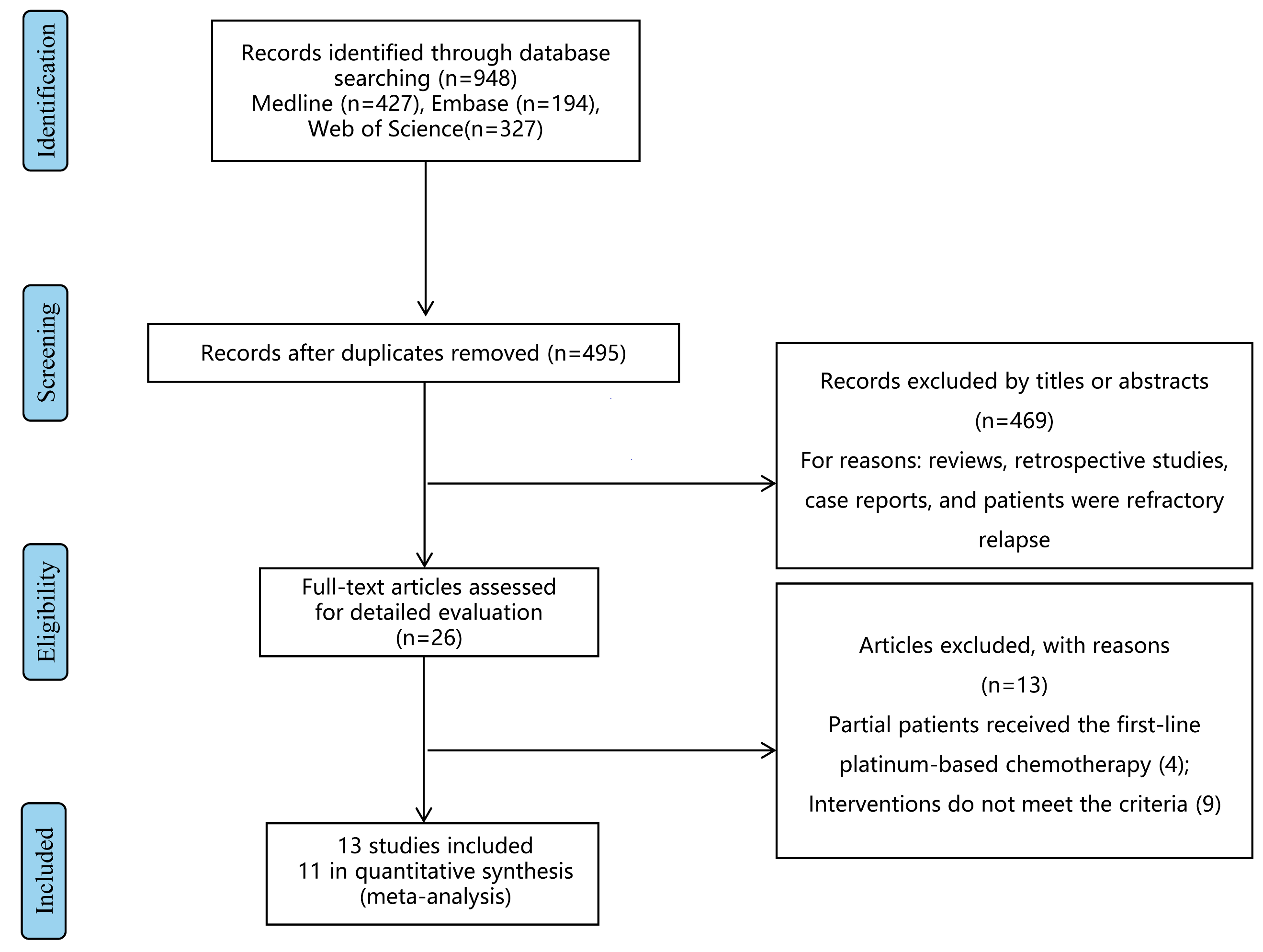 |
| --- |
